# Use of a Google Map Tool Embedded in an Internet Survey Instrument: Is it a Valid and Reliable Alternative to Geocoded Address Data?

**DOI:** 10.2196/resprot.2946

**Published:** 2014-04-10

**Authors:** Sharoda Dasgupta, Adam S Vaughan, Michael R Kramer, Travis H Sanchez, Patrick S Sullivan

**Affiliations:** ^1^Department of EpidemiologyRollins School of Public HealthEmory UniversityAtlanta, GAUnited States

**Keywords:** HIV, geographic mapping, survey, validity, reliability

## Abstract

**Background:**

Men who have sex with men (MSM) in the United States are at high risk for human immunodeficiency virus (HIV) and poor HIV related outcomes. Maps can be used to identify, quantify, and address gaps in access to HIV care among HIV-positive MSM, and tailor intervention programs based on the needs of patients being served.

**Objective:**

The objective of our study was to assess the usability of a Google map question embedded in a Web-based survey among Atlanta-based, HIV-positive MSM, and determine whether it is a valid and reliable alternative to collection of address-based data on residence and last HIV care provider.

**Methods:**

Atlanta-based HIV-positive MSM were recruited through Facebook and from two ongoing studies recruiting primarily through venue-based sampling or peer referral (VBPR). Participants were asked to identify the locations of their residence and last attended HIV care provider using two methods: (1) by entering the street address (gold standard), and (2) “clicking” on the locations using an embedded Google map. Home and provider addresses were geocoded, mapped, and compared with home and provider locations from clicked map points to assess validity. Provider location error values were plotted against home location error values, and a kappa statistic was computed to assess agreement in degree of error in identifying residential location versus provider location.

**Results:**

The median home location error across all participants was 0.65 miles (interquartile range, IQR, 0.10, 2.5 miles), and was lower among Facebook participants (*P*<.001), whites (*P*<.001), and those reporting higher annual household income (*P*=.04). Median home location error was lower, although not statistically significantly, among older men (*P*=.08) and those with higher educational attainment (*P*=.05). The median provider location error was 0.32 miles (IQR, 0.12, 1.2 miles), and did not vary significantly by age, recruitment method, race, income, or level of educational attainment. Overall, the kappa was 0.20, indicating poor agreement between the two error measures. However, those recruited through Facebook had a greater level of agreement (κ=0.30) than those recruited through VBPR methods (κ=0.16), demonstrating a greater level of consistency in using the map question to identify home and provider locations for Facebook-recruited individuals.

**Conclusions:**

Most participants were able to click within 1 mile of their home address and their provider’s office, and were not always able to identify the locations on a map consistently, although some differences were observed across recruitment methods. This map tool may serve as the basis of a valid and reliable tool to identify residence and HIV provider location in the absence of geocoded address data. Further work is needed to improve and compare map tool usability with the results from this study.

## Introduction

### Internet-Based Questionnaires

Internet-based questionnaires have become more popular as a mode of data collection because of the expansive connectivity of individuals in the United States to the Internet overall, and across different social strata, such as education and income [1-3]. Internet-based sampling and data collection have become more practical over the years, as they have the potential to increase responses rates through improved convenience of participation, improved quality of data through programmed validated checks, and decreased costs associated with printing and postage in sending paper-based questionnaires [4,5].

In the context of human immunodeficiency virus (HIV) research, Web-based surveys may provide a sense of anonymity for men who have sex with men (MSM) in the United States, potentially reducing misclassification bias for sensitive questions pertaining to risky sexual behavior and history of HIV testing, diagnosis, and care engagement patterns [4]. Internet recruitment is also an attractive option in studies of MSM who are at high risk for HIV or poor HIV related outcomes. In a longitudinal study conducted by author PSS, 432/483 (89.4%) Atlanta-based MSM, recruited primarily through venue-based sampling, reported using Facebook, Twitter, or another social media site in the previous six months; 328/483 (67.9%) accessed such sites at least once a day (oral personal communication with Nicole Luisi, MPH, and Eli Rosenberg, PhD, July 2013). Further, a meta-analysis showed that an estimated 40% of MSM in the United States reported finding sex partners on the Internet [6]. Finding partners on the Internet may be associated with an increased risk of unprotected anal intercourse [6-8], and potentially sexually transmitted infections such as HIV [9].

### Geographic Information Systems

Recently, there has been a growing emphasis on how certain contextual factors can affect disease; specifically, trends in disease incidence or prevalence, or potential predictors of poor outcomes, may vary based on an individual’s environment or setting. Quantifying such differences across neighborhoods and community level factors using a geographic information systems (GIS)-based approach can impact public health programs and policy [10,11]. In the context of HIV, maps may be important in discerning hot spots of disease, high risk behaviors, such as illicit drug use and unprotected sex, and the level of health care access and utilization once diagnosed. GIS analyses can also examine how certain neighborhood level characteristics affect the dynamics of HIV transmission and patterns in HIV care engagement, which may lead to tailored interventions based on individual characteristics and needs [12,13]. For instance, using maps to quantify accessibility (in relation to proximity to different health services) may be helpful in identifying and addressing gaps in access to care, and tailoring intervention programs based on the needs of the patients being served [14].

However, map tools embedded in Internet surveys used to identify key locations for study participants are not widely used currently, and to our knowledge, have never been evaluated for usability in the context of validity and reliability. In the absence of address data, the development of a valid and reliable map tool to identify key locations, such as patient’s residence and location of the patient’s HIV care provider office, may be important in quantifying place-based barriers to care attendance, including travel distance and proximity to public transportation. Alternatively, such a map tool can be used to identify where people test for HIV or might be finding high risk sex partners (it may not be easy to obtain a valid address for the latter location). Even given a known address, however, automated geocoding systems vary in the degree of accuracy, result in nonnormally distributed errors, and may be less accurate outside of urban areas [15,16].

We conducted a cross-sectional study of Atlanta-based, HIV-positive MSM, called “The Engage Study”, to explore potential place-based barriers to care, including proximity to HIV care provider and neighborhood level characteristics, such as socioeconomic status (SES). In this analysis, we assess the level of usability of a Google map question embedded in a Web-based survey, and determine whether it is a valid and reliable alternative to a geocoded address in identifying residence and last attended HIV care provider.

## Methods

### Study Population

The Engage Study is a cross-sectional study of self-identifying HIV-positive MSM living in the Atlanta area. The study was designed to examine potential structural and psychosocial barriers to accessing HIV care and treatment. Men were recruited from October 2012 to June 2013 through two sources: (1) based on participation in prior studies of MSM conducted by Emory University, and (2) from Facebook.

Men documented to be HIV-positive through HIV testing in two other Emory-based studies of MSM were recontacted by phone and email for participation in The Engage Study. These two prior studies aimed to examine racial/ethnic differences in black and white HIV epidemics in Atlanta, so only black and white MSM were eligible to participate in these two studies. Participants from the two studies were originally recruited in Atlanta, primarily through venue-based sampling or peer referral (VBPR). Men who agreed to participate in the present study were sent an email with a link that directed them to the eligibility screener and informed consent form.

Facebook advertisements for the study were targeted toward men who were interested in other men and lived within 50 miles of Atlanta. Those who clicked on the advertisement were directed to the survey and presented with a Web-based eligibility screener (including assessment of self-reported HIV status) and informed consent form. Men recruited from Facebook were not restricted by race. However, since very few Facebook-recruited participants reported another race, those who did not identify as black or white were excluded from all analyses to avoid sparsely populated data by race and maintain comparable groups across recruitment method.

Men from both methods of recruitment were deemed eligible to participate in the present study if they reported being at least 18 years of age, ever having sex with another man, being told they are HIV-positive by a health care provider, and currently living in the Atlanta area. All consenting participants were directed to the one-time, Web-based survey instrument, administered using the Internet survey software platform, SurveyGizmo [17]. Participants were asked to take the survey on a personal computer or tablet to minimize issues with the display and layout of the questionnaire that might have occurred on a smartphone or simple mobile device. The Emory University Institutional Review Board approved the protocol (approval number, IRB00060430).

### Measures

The questionnaire collected detailed information on demographic characteristics, where participants lived and sought HIV care and potential structural and psychosocial barriers to HIV care engagement, such as transportation, travel distance, and HIV related stigma. For two key locations, their home and the provider or clinic where they last received HIV care, respondents were asked to provide location information in two ways: (1) using a text address field (or name of the provider or clinic, to allow research staff to find the street address), and (2) by clicking on the location in the Google map embedded in the survey.

For their residential location, participants first entered address data using text fields for street address, city, state, and zip code. Next, they were asked to click on their residential location on a Google map embedded in the survey. For the HIV care provider location, respondents first selected from a checkbox menu of providers located in, or close to, Atlanta, based on information from the Southeast AIDS Training and Education Center (SEATEC) Key Contacts booklet [18]. Addresses for each of these HIV care providers were also available in the SEATEC booklet. If participants reported receiving care from a provider outside of the SEATEC network (eg, a private infectious diseases provider practice), they were asked to report the name of their doctor and the address or area of town where his or her office was located. The research staff then determined the exact address of the provider’s office. Next, participants clicked on the approximate location of their provider’s office using the Google map.

**Figure 1:**
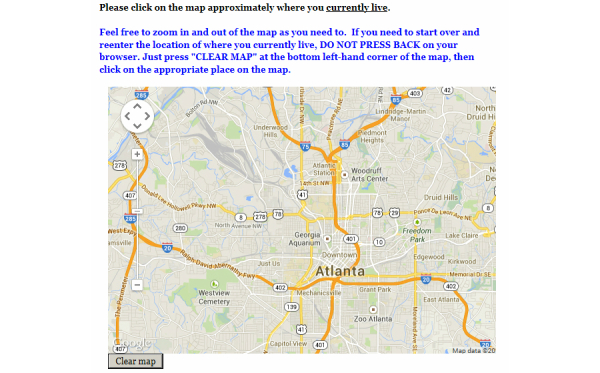
A screenshot of a survey question used to identify, using the Google map, the location of residence among a convenience sample of HIV-positive men who have sex with men, Atlanta, Georgia, 2012-2013. A similar survey question was used to identify the location of each participant’s HIV health provider’s office.

Detailed instructions on how to specify a location on the map were provided in each map question. Address and map derived locations were collected independently (ie, the physical address provided in one section did not change the initial map focus for the map derived location). The map allowed the user to zoom in as much as needed to click on the appropriate location, but the initial zoom level allowed users to view major streets in Atlanta (approximately 1:127,000, or an inch representing approximately 2 miles). Participants had the option to clear the map and click on another point on the map, if they incorrectly identified the location. Figure 1 shows a screen shot of this survey question.

### Analytic Methods

Using the residential address and the address of the attended HIV care provider or clinic as gold standards, we assessed the validity of using map derived information by examining how the locations specified using map-based technology differed from the gold standard locations. Further, to assess reliability, we examined the consistency in the level of error in identifying residential location versus the location of the most recent HIV care provider. All analyses were restricted to participants who were of black or white race, did not report being homeless at the time of the survey, and lived more than 50 miles away from the center of Atlanta, and were conducted using ArcGIS 10.1 and SAS 9.3.

### Descriptive Statistics

Descriptive statistics for demographic characteristics of respondents were computed using counts and frequencies. Differences in demographic characteristics were evaluated across recruitment method using the Mantel-Haenszel chi-square test.

### Analysis of Validity

**Figure 2:**
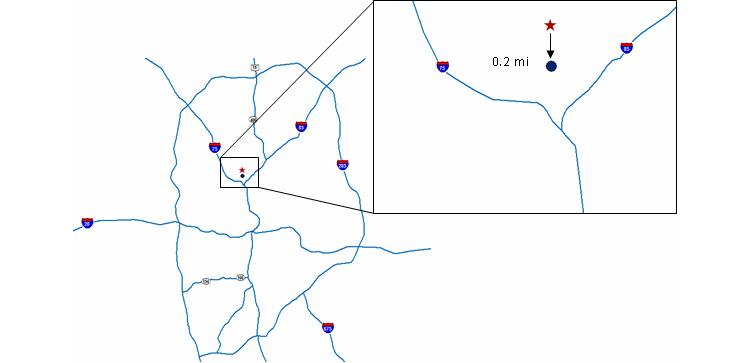
A visual example of how home and provider location errors were calculated among The Engage Study participants in Atlanta, Georgia. The starred point represents the gold standard, geocoded address, and the dot is where the participant perceived the location to be on the map. Geodesic distance (the shortest distance between two points, or “as the crow flies”) was then computed between these two points.

Respondents’ text-based residential and HIV care provider addresses were geocoded (defined by geographic coordinates corresponding to address data) [19] using ArcGIS 10.1. Geocoded addresses and the latitude and longitude coordinates corresponding to the clicked map points for both the residence and HIV care provider were then mapped (using the North American Albers Equal Area Conic projection) [19], and geodesic distances (the shortest distance between two points on a sphere or curved surface) between the address data (“the gold standard”) and the clicked map coordinates were calculated. These geodesic distances represent the “error” between the gold standard location and where participants identified them to be on a Google map. Figure 2 shows a visual example of this calculation. To distinguish between the residential and HIV care provider validation analyses in this paper, we will refer to the comparison of map-based versus gold standard locations for patient’s residence as home location error, and the comparison of map-based versus gold standard locations for last attended HIV care provider as provider location error.

We computed descriptive statistics for the two primary outcomes (home and provider location errors) using medians and interquartile ranges (IQR), because both were continuous, but nonnormally distributed. Because we hypothesized, a priori, that participants have a greater Internet literacy and might be better able to navigate through the map questions, we assessed differences in the home and provider location errors across both recruitment method and demographic characteristics using the Wilcoxon-Mann-Whitney test. An alpha cutoff of 0.05 determined statistical significance.

### Analysis of Reliability

To examine whether home and provider location errors were consistent within participants, the provider location error values were plotted against home location error values; results were stratified by recruitment method. Further, geodesic distances were dichotomized as either less than one mile or at least one mile, and a kappa statistic was computed to assess “agreement” or “reliability” of degree of error in identifying the patient’s residential location versus the HIV care provider location. A kappa statistic less than or equal to 0.20 indicated poor agreement, 0.21-0.40 indicated fair agreement, 0.41-0.60 indicated moderate agreement, and greater than 0.60 indicated substantial agreement [20] of degree of error in identifying the residential location versus the provider location. In this portion of the analysis, we hypothesized, a priori, that the residential and provider location errors are consistent within each participant and expect at least fair to moderate agreement between the two measures.

## Results

### Descriptive Statistics

Out of the 293 HIV-positive, VBPR-recruited MSM who agreed to be recontacted for other research studies, 131 (44.7%) participated. Approximately 40,000 Facebook users were targeted for recruitment based on the criteria described above, out of which 82 (0.21%) met the eligibility criteria and participated in the study. Thus, a total of 213 self-identifying HIV-positive MSM participated in The Engage Study. There were 3 (1.4%) participants that were excluded from all analyses because they lived more than 50 miles outside Atlanta. For the home location error analysis, 35/210 (16.7%) participants were further excluded because they did not respond to the map-click question to identify their residential location, 27/210 (12.9%) participants were excluded because they did not report a valid text version of the home address, and 6/210 (2.9%) participants were excluded because both of these were missing. Thus, out of 210 participants living in the Atlanta area, 142 (67.6%) were included in the patient’s home error analysis. For the HIV care provider location error analysis, 8/210 (3.8%) participants were excluded because they reported never receiving HIV care, 33/210 (15.7%) participants were excluded because they did not complete the map click question to identify their provider location, 12/210 (5.7%) participants were excluded because they listed a provider whose office location could not be geocoded, and 3/210 (1.4%) participants were excluded because neither provider address nor clicked points were available. As such, 154/210 (73.3%) respondents were included in the HIV care provider error analysis. A total of 112 (53.3%) out of the 210 participants living within 50 miles of Atlanta completed all four questions related to their home and HIV care provider locations and were used in the analysis of reliability. 

The distribution of demographic characteristics of study participants is described in Table 1. Median age of participants was 34 years old. Almost half of the participants reported an annual household income of less than US $20,000, a majority of participants reported being of black/African American race, and about a third of the sample reported having a college degree. Demographic characteristics varied across method of recruitment.

**Table 1:** Demographic characteristics and error distances among a convenience sample of HIV-positive MSM, overall and by recruitment method, Atlanta, Georgia, 2012-2013.

		All participants (N=210)	Internet recruitment (n=81)	VBPR recruitment (n=129)	*P* ^b^
		number (%)^a^	number (%)^a^	number (%)^a^	
**Age**					<.001
	18-35 years	117 (55.7)	24 (29.6)	93 (72.1)	
	> 35 years	93 (44.3)	57 (70.4)	36 (27.9)	
**Race**					<.001
	White/Caucasian	73 (36.9)	45 (63.4)	28 (22.0)	
	Black/African American	125 (63.1)	26 (36.6)	99 (78.0)	
**Household income**					<.001
	< US $20,000 / year	111 (54.4)	31 (38.8)	80 (64.5)	
	> US $20,000 / year	93 (45.6)	49 (61.2)	44 (35.5)	
**Education**					.06
	High school education or less	41 (19.7)	14 (17.5)	27 (21.1)	
	Some college, associate's degree, and/or technical school	99 (47.6)	33 (41.3)	66 (51.6)	
	College, post graduate, or professional school	68 (32.7)	33 (41.3)	35 (27.3)	
**Location type** ^c^					
	Home location error	142 (67.6)	63 (77.8)	79 (61.2)	.01
	Provider location error	154 (73.3)	57 (70.4)	97 (75.2)	.44

^a^Whole percentages may not sum to 100 due to rounding. Numbers may not sum up to total because of missing values.

^b^Mantel-Haenszel chi-square test was used to determine statistical significance.

^c^These rows indicate counts of data available to calculate the patient’s home and the HIV care provider location errors.

### Analysis of Validity

**Figure 3:**
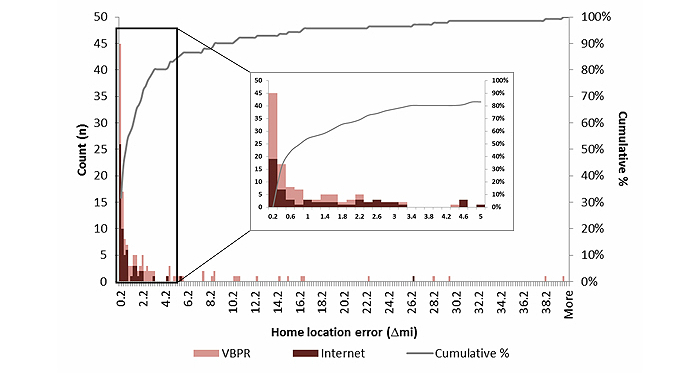
The probability density function and cumulative distribution function of home location error among a convenience sample of HIV-positive men who have sex with men who reported their home address and identified the location on a map (n=142) by recruitment mode, Atlanta, Georgia, 2012-2013. ∆mi=geodesic distance between geocoded location of home address and where participants identified their home on the map. VBPR=venue-based sampling or peer referral.

Out of 142 participants included in the home location error analysis, 80 (56.3%) clicked within a mile of their home address; however, a greater proportion of Facebook-recruited individuals clicked within a mile of their reported residential address, compared to VBPR-recruited participants (47/63, 74% vs 33/79, 41%; *P*<.001). Figure 3 shows a detailed plot of the distribution of home location error by recruitment method. The median home location error across all participants was 0.65 miles (IQR, 0.10, 2.5 miles), but was also significantly higher among VBPR participants (*P*<.001), as well as among blacks (*P*<.001), and those reporting lower annual household income (*P*=.04). Younger age (*P*=.08) and lower educational attainment (*P*=.05) were also associated with greater median home location error, but not statistically significantly (Table 2).

**Figure 4:**
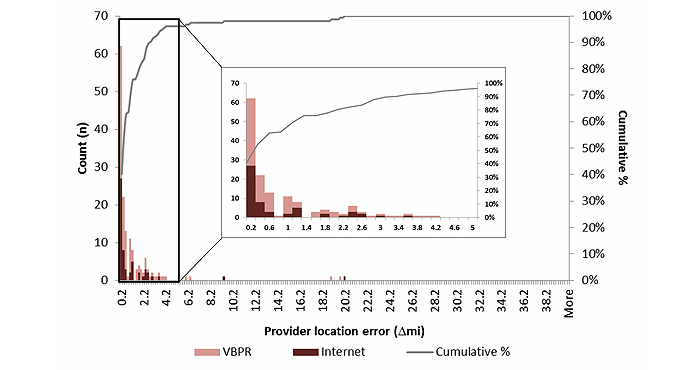
The probability density function and cumulative distribution function of provider location error among a convenience sample of HIV-positive men who have sex with men who reported where they last received HIV care and identified the location on a map (n=154) by recruitment mode, Atlanta, Georgia, 2012-2013. ∆mi=geodesic distance between geocoded location of the HIV care provider address and where participants identified their provider on the map. VBPR=venue-based sampling or peer referral.

Out of 154 participants included in the provider location error analysis, 109 (70.8%) clicked within a mile of their HIV care provider. Figure 4 shows a detailed plot of the distribution of provider location error by recruitment method. The median provider location error was 0.32 miles (IQR, 0.12, 1.2 miles), and did not vary significantly by recruitment method, race, income, or level of educational attainment. Although not statistically significant (*P*=.06), the median provider location error was notably lower among older participants compared to younger participants (0.46 miles compared to 0.23 miles) (Table 2).

**Table 2:** The level of the patient’s home and HIV care provider location error among a convenience sample of HIV-positive MSM by demographic characteristics and recruitment mode, Atlanta, Georgia, 2012-2013.

		Home location error (miles)			Provider location error (miles)		
		Median	IQR	*P* ^a^	Median	IQR	*P* ^a^
Overall		0.65	(0.10, 2.47)		0.32	(0.12, 1.15)	
**Age**				.08			.06
	18-35 years	0.79	(0.11, 5.56)		0.46	(0.12, 1.74)	
	> 35 years	0.56	(0.08, 1.95)		0.23	(0.11, 1.02)	
**Race**							.58
	White/Caucasian	0.20	(0.05, 0.68)	<.001	0.26	(0.11, 1.00)	
	Black/African American	1.53	(0.30, 5.02)		0.32	(0.11, 1.15)	
**Household income**				.04			.92
	< US $20,000	1.22	(0.15, 5.34)		0.39	(0.11, 1.15)	
	> US $20,000	0.44	(0.08, 1.55)		0.30	(0.12, 1.34)	
**Education**				.05			.55
	High school or less	1.28	(0.17, 4.43)		0.36	(0.12, 2.12)	
	Some college, associate’s degree, or technical school	0.85	(0.16, 4.77)		0.34	(0.11, 1.05)	
	College, post graduate, or professional school	0.35	(0.08, 1.33)		0.19	(0.11, 1.07)	
**Recruitment mode**				<.001			.31
	VBPR	1.71	(0.24, 5.34)		0.38	(0.12, 1.51)	
	Internet	0.30	(0.06, 1.17)		0.29	(0.11, 1.12)	

^a^Wilcoxon-Mann-Whitney test was used to determine statistical significance.

### Analysis of Reliability

**Figure 5:**
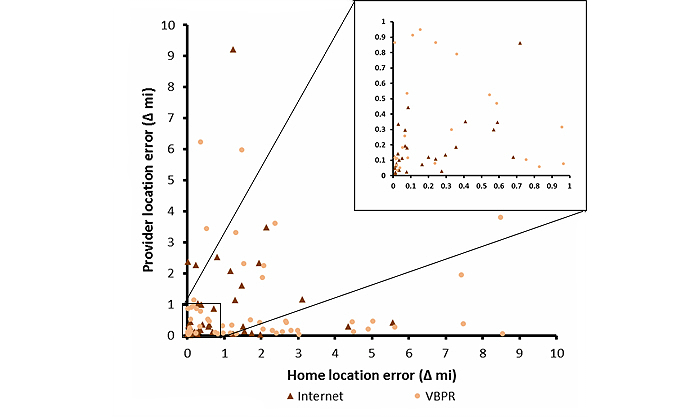
Plot of the home location error versus the provider location error among a convenience sample of HIV-positive men who have sex with men who answered all four location questions (n=112), coded by recruitment method; Atlanta, Georgia, 2012-2013. ∆mi=geodesic distance between geocoded location of the HIV care provider address and where participants identified their provider on the map. VBPR=venue-based sampling or peer referral.

The plot of the provider location error against home location error in Figure 5 illustrates that, among VBPR-recruited individuals, the spread of the home location error (range, 39.8 miles) is much greater than that of the provider location error (range, 19.7 miles). Further, the range of errors overall were smaller and more consistent among Facebook participants (home location error range, 26.2 miles; provider location error range, 20.1 miles), compared to VBPR participants. Only 23/64 (35%) VBPR participants clicked within a mile of the gold standard locations for their residence and HIV care provider, while 27/48 (56%) Facebook-recruited participants clicked within the same distance of the gold standard locations. R-squared values were calculated to measure the correlation between home location error and provider location error, but were not significant, and therefore are not reported. Because the plot of the two error measures was restricted to observations for which all four location-based questions were answered, we examined, in a post-hoc analysis, the demographic characteristics of those who answered all four questions versus those who did not, to address any potential concerns related to selection bias. There were no statistically significant differences in age, race, income, or educational attainment across these two groups.

A simple kappa coefficient was also computed to assess the level of reliability between home location error and provider location error. Overall, the kappa statistic was 0.20, bordering on poor to fair agreement between the two error measures. However, those recruited through Facebook had a greater agreement (κ=0.30) than those recruited through VBPR methods (κ=0.16), demonstrating a greater level of consistency in using the map question to identify the patient’s home and the HIV care provider locations for Facebook-recruited individuals.

## Discussion

### Principal Results

In this study, we aimed to assess, among a convenience sample of Internet-using, Atlanta-based, HIV-positive MSM, the validity (ie, the degree of error between map derived location information and the gold standard) and reliability (ie, the consistency in the degree of error in locating the patient’s home vs the HIV care provider location) of using a Google map question embedded in an Internet survey instrument to identify the patient’s residential and the HIV care provider location, as compared to the geocoded address information. The results demonstrate the map tool’s validity, as a majority of the study participants were able to click within a mile of their home and most recently attended HIV care provider. However, the reliability and usability varied by recruitment method.

Although most participants were able to click within a mile of their residence, there were observed differences in home location error by recruitment method and markers of SES, such as race and household income, which may be attributed to differences in the intensity of Internet use among participants. Though a majority of US residents have access to the Internet [2,21], population-based estimates in the United States show that Internet use varies by race, education, and income [2,22]. Specifically, blacks, those with lower educational attainment and those reporting a lower household income, are less likely to report using the Internet either at home or elsewhere [22,23]. Further, by 2012, nearly half of all Americans reported owning a smartphone, a potential indicator of the level of connectivity to the Internet through multiple devices. This proportion is lower among those reporting a lower household income and lower educational attainment, suggesting possible differences in the level of connectivity and intensity of Internet use across markers of SES [24]. Participants recruited through Facebook in the present study were more likely to be older, be of white race, report a higher annual household income, and report a higher level of educational attainment, and thus, may have had greater connectivity to the Internet than VBPR participants.

Higher intensity of Internet use, especially through multiple devices, may also be associated with a greater ability to navigate through the mapping questions successfully. In addition, eligible Facebook users who check their accounts more frequently may have been more likely to view the recruitment advertisement, and thus, may have been more likely to click through the advertisement and participate. Therefore, Facebook-recruited participants, who were more likely to accurately and consistently identify their residence and provider’s office, may also have been more frequent Internet users and, therefore, more likely to be able to navigate through an interactive, Internet-based mapping tool.

Observed differences in consistency by recruitment method may also be explained by the order in which the mapping questions were presented in the survey. The map asking participants to identify their residence was shown first in the questionnaire, whereas the HIV care provider map was presented later on in the survey. If participants were more likely to have trouble initially orienting themselves to the mapping questions, but became accustomed to the format of the question for the HIV care provider map, there may have been a “learning effect”, resulting in a higher patient’s home location error and a lower HIV care provider location error. Conversely, those individuals already accustomed to using Google maps may have been more consistently and accurately able to identify both residence and place of care in the survey. This may be why a greater level of consistency was observed among Facebook-recruited participants, if they are more frequent Internet users than VBPR-recruited individuals. It may also be important to note that the zoom level on each map question was not fixed. The participant could zoom in and out as needed to identify each location; therefore, those who utilized the zoom level may have been more likely to click closer to the gold standard location than those who did not. Again, perhaps frequency of Internet use may be associated with the level of comfort and usability of the mapping question format and zoom feature, which may explain why this “learning effect” trend may have been observed to a lesser extent among Facebook-recruited participants.

### Limitations

There are several limitations to this analysis. First, we recruited a convenience sample that may not be representative of the target population of Internet-using, HIV-positive MSM in Atlanta. Homeless individuals were excluded from the analysis, further limiting generalizability of the results. Even among those recruited, a large proportion of participants did not provide both map-based location and address data for their home and HIV care provider, respectively, and thus, had to be excluded from the validation analysis.  The reliability analysis was underpowered, as almost half of the participants did not complete all four questions related to residential and care provider locations.  There is also a potential for selection bias in excluding participants in the reliability analysis, but no statistically significant differences in age distribution, race, annual household income, and level of educational attainment were observed between those who answered both location-based questions for the patient’s residence and the HIV care provider’s office and those who did not. The reasons for not answering these survey questions should be further explored by convening a small post test focus group.

In addition, the zoom level at which participants clicked on the map questions was not recorded during data collection, which may be associated with the level of accuracy of clicked map points in relation to the gold standard location. Either implementing a fixed zoom level or capturing information on the zoom level used for each participant would be helpful in controlling for any potential variability caused by this factor. The usability of the map tool could vary by the type of device used to take the questionnaire, but information on the exact device type used was not collected, and participants were encouraged to take the survey on a personal computer or tablet instead of a phone. Future studies should highlight device type as a potential source of variation in usability, validity, and reliability of a map-based tool.

Last, one minor limitation of using geodesic distances as a metric for assessing home and provider location error is that they may actually underrepresent the difference in actual distance between the map-based locations and the address data. Further, for subsequent neighborhood level analyses using these data, even small values for home or provider location errors may point to a different neighborhood with different community level characteristics from those of the gold standard location.

Despite these limitations, future studies incorporating improvements to the zoom level, information on the device type used, feedback from pre and post test focus groups, and training sessions to assess feasibility and usability could add to the results from this study and existing knowledge on the usability of a map tool to evaluate location-based information.

### Conclusions

Despite the observed differences in the patient’s home and the HIV care provider location errors across certain markers of SES and recruitment method, the map tool proved to be a valid alternative to geocoded addresses, as most participants were able to click within a mile of their home address and their HIV care provider’s office. However, the tool bordered on poor to fair reliability between home location error and the HIV care provider location error, although those recruited on the Internet generally had better agreement, or consistency, between their home and HIV care provider location errors.

Although there are improvements to be made in this map tool, it may serve as the basis for a valid and reliable tool to identify important locations in the absence of geocoded address data. The limitations in the usability of the tool should be addressed by offering a short training session for participants prior to taking the survey. Other problems related to the layout, functionality, or usability of the tool can also be identified and addressed in a small focus group. An improved version of this Google map-based survey question can be used to capture important data on health care utilization and neighborhood level risk factors for poor health outcomes, which can have important implications in intervention planning.

## Abbreviations

GIS: geographic information systems
HIV: human immunodeficiency virus
IQR: interquartile range
MSM: men who have sex with men
SEATEC: Southeast AIDS Training and Education Center
SES: socioeconomic status
VBPR: venue-based sampling or peer referral
